# Bacterial Sub-Species Typing Using Matrix-Assisted Laser Desorption/Ionization Time of Flight Mass Spectrometry: What Is Promising?

**DOI:** 10.3390/cimb43020054

**Published:** 2021-07-20

**Authors:** Charlotte A. Huber, Sarah J. Reed, David L. Paterson

**Affiliations:** University of Queensland Centre for Clinical Research, The University of Queensland, Herston, Brisbane, QLD 4029, Australia; sj.reed@uq.edu.au (S.J.R.); d.paterson1@uq.edu.au (D.L.P.)

**Keywords:** MALDI-TOF MS typing, MALDI-TOF MS, bacterial sub-species typing, strain typing, bacterial strain identification

## Abstract

Matrix-assisted laser desorption ionization–time of flight mass spectrometry (MALDI-TOF MS) is routinely used for bacterial identification. It would be highly beneficial to also be able to use the technology as a fast way to detect clinically relevant clones of bacterial species. However, studies to this aim have often had limited success. The methods used for data acquisition, processing and data interpretation are highly diverse amongst studies on MALDI-TOF MS sub-species typing. In addition to this, feasibility may depend on the bacterial species and strains investigated, making it difficult to determine what methods may or may not work. In our paper, we have reviewed recent research on MALDI-TOF MS typing of bacterial strains. Although we found a lot of variation amongst the methods used, there were approaches shared by multiple research groups. Multiple spectra of the same isolate were often combined before further analysis for strain distinction. Many groups used a protein extraction step to increase resolution in their MALDI-TOF MS results. Peaks at a high mass range were often excluded for data interpretation. Three groups have found ways to determine feasibility of MALDI-TOF MS typing for their set of strains at an early stage of their project.

## 1. Introduction

Genotyping methods often have good discriminatory power to track bacterial strains but tend to be costly and time consuming. Whole genome sequencing (WGS), in particular, is a very powerful tool for the investigation of phylogenetic relationships and is becoming the gold standard for this purpose. However, in addition to the costs that are still high for data acquisition, considerable bioinformatics expertise is required for data analysis of WGS [1,2].

MALDI-TOF MS is routinely used in clinical laboratories for identification of bacterial species. It would be of great interest to be able to use existing MALDI technology to identify clinically relevant clones of a bacterial species in a timely manner to help infection control management.

However, studies to this aim are conflicting about the usefulness of MALDI-TOF MS for bacterial strain identification [3]. Challenges may arise because of differences between bacterial strains being smaller than between bacterial species. MALDI-TOF MS results may fluctuate due to differences in bacterial growth conditions, sample preparation or matrix used, and bacterial strain typing may be more susceptible to such fluctuations than bacterial identification [4].

There is a lack of standardisation amongst published studies, where MALDI-TOF MS data have been interpreted by varying bioinformatics tools, and in some cases manually. Additionally, the feasibility of MALDI-TOF MS typing may depend on the bacterial species or strains investigated, making it difficult to determine what methods make MALDI-TOF MS typing work [5,6].

In our paper, we review publications on MALDI-TOF MS typing of bacterial isolates of several bacterial species as a fast and inexpensive way to identify bacterial strains [6]. The publications were selected based on overall diversity of bacterial species investigated, sample size and the use of genotyping reference methods for comparison.

## 2. Approaches Taken by Research Groups for the MALDI-TOF MS Typing of Bacterial Isolates

### 2.1. MALDI-TOF MS Typing of Staphylococcus aureus Isolates

Wang et al. typed 306 methicillin-resistant *Staphylococcus aureus* (MRSA) isolates using MALDI-TOF MS. Data for strain distinction were acquired using a Bruker MicroFlex LT mass spectrometer and analyzed with the help of machine learning algorithms of the ClinProTools software (Bruker Daltonik GmbH, Bremen, Germany). Ten peaks ranging from 2082 to 6594 Da were selected for discrimination, and the results were compared with those obtained from the multilocus sequence typing (MLST) method of the respective isolates.

The experiments were performed as follows: Bacterial isolates were grown for 24 h, colonies were spotted and overlaid with formic acid and after drying, alpha-cyano-4-hydroxycinnamic acid (CHCA)-based matrix (1% CHCA in 50% acetonitrile and 2.5% trifluoroacetic acid) was added. Mass spectra were acquired in the range from 2000–20,000 Daltons (Da) using a Bruker MicroFlex LT mass spectrometer. The MALDI-TOF mass spectra were imported into ClinProTools^TM^. Ten peaks ranging from 2082 to 6594 Da were selected for discrimination of isolates of differing MLST types, taking peak intensities into account. Spectra of isolates with any of the three most prevalent MLST types (ST5, *n* = 40; ST59, *n* = 62; ST239 *n* = 179) were classified using machine learning algorithms of ClinProTools.

The authors found that in the case of these isolates, MALDI-TOF MS spectra could successfully be used to predict the respective MLST type [6].

Steensels et al. used MALDI-TOF MS to type 20 MRSA isolates which included 11 outbreak-related isolates, four outbreak-unrelated isolates and five reference strains. Data for strain distinction were acquired using a Bruker Microflex LT MALDI-TOF MS instrument and interpreted based on pattern similarity using the BioNumerics software (Applied Maths, Sint-Martens-Latem, Belgium). The results were compared with the results of pulsed field gel electrophoresis (PFGE) and *Staphylococcus aureus* protein A (*spa*) typing.

The experiments were performed as follows: Isolates were grown overnight and subjected to ethanol–formic acid extraction. One microlitre of extract was spotted eight times and overlaid with CHCA-based matrix (CHCA in 50% acetonitrile and 2.5% trifluoroacetic acid). Each spot was measured three times on a Bruker Microflex LT MALDI-TOF MS instrument in the mass range of 2000–20,000 Da. The resulting spectra were imported to the BioNumerics software, and a mean spectrum profile was created for each isolate. Dendrograms were generated based on similarity comparison of the fingerprint patterns of the mean spectra. MALDI-TOF MS types were assigned using a cut-off value of more than 95% similarity.

A 93% agreement was achieved between MALDI-TOF MS typing and PFGE as well as *spa* typing. Out of the 11 outbreak isolates included in the study, 10 displayed an identical MALDI-TOF MS type that was distinct from non-outbreak types. The study showed that MALDI-TOF MS typing may be helpful for outbreak management. However, the reproducibility of MALDI-TOF MS typing was poor, limiting its practicability for routine use [7].

Østergaard et al. used MALDI-TOF MS to subtype 378 MRSA isolates of the commonly livestock-associated clonal complex 398. Data for strain distinction were acquired using a Bruker MALDI-TOF MS instrument, and the isolates were typed based on 17 marker peaks in the range of 4446 to 9361 m/z. The results were compared with the results of single nucleotide polymorphism typing of the respective whole genome sequences.

The experiments were performed as follows: Bacterial isolates were streaked directly from overnight cultures, overlaid with CHCA-based matrix (saturated CHCA solution in 50% acetonitrile and 2.5% trifluoroacetic acid) and measured in a Bruker MALDI-TOF MS instrument. This was repeated at a later stage to test reproducibility. All mass spectra were internally calibrated using a peak of 4306 m/z which was present in all isolates. Seventeen marker peaks in the range of 4446 to 9361 m/z could be used to reproducibly divide the isolates into 23 subtypes.

The authors state that whilst their MALDI-TOF typing method was not very good at predicting close phylogenetic relationships, isolates of different MALDI subtypes were unlikely to be closely related. Therefore, isolates of different MALDI subtypes could with high certainty be excluded from being outbreak related [2].

### 2.2. MALDI-TOF MS Typing of Klebsiella Isolates

Dinkelacker et al. investigated 68 *Klebsiella* isolates, using MALDI-TOF MS typing and WGS. Data for strain distinction were acquired using a Bruker MALDI biotyper system and interpreted based on spectrum similarity, using the Bionumerics software suite. The MALDI-TOF MS typing results were compared against the results of single nucleotide polymorphism typing of the core genome of each respective isolate.

The experiments were performed as follows: Each isolate was spotted in quadruplicate in three independent experiments and overlaid with CHCA-based matrix (CHCA in 50% acetonitrile and 2.5% trifluoroacetic acid). The samples were measured on a Bruker MALDI biotyper system in a mass range of 2000 to 20,000 Da. The data were imported into the Bionumerics software suite for processing. The four spectra of each of the three experiments were used to build a summary spectrum and an isolate consensus spectrum was then generated out of the three summary spectra. A curve-based or peak-based similarity matrix was then calculated.

The congruence between WGS typing and MALDI-TOF MS typing was found to be poor, however, independent of the clustering algorithm chosen [8].

Angeletti et al. tested 25 carbapenem-resistant *Klebsiella pneumoniae* isolates using MALDI-TOF MS. Data for strain distinction were acquired using a Bruker MALDI biotyper and interpreted with the help of the ClinProTools software (Bruker Daltonik GmbH, Bremen, Germany). Ten peaks ranging from 4154–9476 Da were used for the comparison of isolate spectra, and the results were compared with MLST and antibiotic susceptibility testing.

The experiments were performed as follows: Bacterial samples were subjected to ethanol–formic acid extraction, spotted and overlaid with CHCA-based matrix (a saturated solution of CHCA in 50% acetonitrile and 2.5% CHCA). The samples were measured in a Bruker MALDI biotyper in the mass range of 2000–20,000 Da. Technical replicate spectra of each isolate were combined into one biological replicate spectrum. A dendrogam of all the biological replicate spectra was generated using Bruker’s ClinProTools software. The 10 peaks with the highest intensity (4154–9476 Da) were used for comparison of the isolate spectra, taking the area under the curve (AUC) of each peak into account.

Two clusters were found with the help of MALDI-TOF MS typing. The clustering could not be confirmed by MLST because most isolates were of the same MLST type. However, some differences could be detected between isolates of the two clusters regarding the time range in which they were collected and their antibiotic resistance patterns [9].

Bar-Meir et al. evaluated the performance of MALDI-TOF MS by typing 33 outbreak-related ESBL-producing *Klebsiella pneumoniae.* Data for strain distinction were acquired using a Bruker Microflex LT MALDI-TOF mass spectrometer and analysed using the MALDI Biotyper 3.0 system. Isolates were discriminated based on peak shifts and the presence or absence of a peak. The results were compared with core genome MLST.

The experiments were performed as follows: Isolates were grown overnight and measured in triplicate using a Bruker Microflex LT MALDI-TOF mass spectrometer. Cluster analysis was performed, and a single main spectrum was created for each isolate, using the MALDI Biotyper 3.0 system (Bruker Daltonics).

Two clones with the following differences could be detected: One clone did not contain a peak at 4100 m/z and had shifts in peaks from 4705 m/z to 4715 m/z and from 4640 m/z to 4660 m/z when compared with the other clone. Isolates of each clone were from patients who lived in different suburbs and were in different hospital rooms. Six isolates representing the two clones were chosen for WGS and compared using core genome MLST. The core genome MLST confirmed the MALDI-TOF MS typing results [1].

### 2.3. MALDI-TOF MS Typing of Clostridium difficile

Reil et al. typed 355 *C. difficile* isolates using MALDI-TOF MS typing. Data for strain distinction were acquired using a Shimadzu AXIMA confidence MALDI-TOF mass spectrometer. Spectra were processed using the BioTech Launchpad software (Shimadzu Europe, Duisburg, Germany) and analysed with the AgnosTec SARAMIS^TM^ software. Several mass peaks ranging from 3361–11,202 m/z were chosen as biomarkers for the distinction between isolates. The results were compared with the results of PCR ribotyping for the respective isolates.

The experiments were performed as follows: The reference database was set up using a standard set of isolates comprising 25 different PCR ribotypes that were part of the European Center for Disease Prevention and Control Brazier (ECDC-Brazier) collection. Isolates were grown under anaerobic conditions for 24 h, spotted and overlaid with CHCA-based matrix (saturated CHCA in 33% ethanol, 33% acetonitrile, 3% triflouroacetic acid and 31% water). Spectra were acquired on a Shimadzu AXIMA confidence MALDI-TOF mass spectrometer. The resulting peak lists were imported into the SARAMIS (bioMérieux) software package for biomarker analysis. SuperSpectra were generated with the AgnosTec SARAMIS^TM^ software, selecting specific biomarkers ranging from 3361–11,202 m/z for the respective ribotypes. The database was validated with 355 *C. difficile* isolates of 29 different PCR ribotypes with the most prevalent ribotypes being 001 (70%), 027 (4.8%) and 078/126 (4.7%). Most of the isolates used for validation were clinical isolates from patients.

Whilst *C. difficile* isolates of more sporadic PCR ribotypes could not be detected, the isolates with the three most frequent PCR ribotypes, 001, 027 and 078/126, could successfully be typed using MALDI-TOF MS [10].

### 2.4. MALDI-TOF MS Typing of Escherichia coli

Veenemans et al. evaluated the discriminatory power of MALDI-TOF MS typing of 52 *E. coli* isolates including outbreak isolates. Data for strain distinction were acquired using a bioMérieux VITEK MS MALDI instrument and analysed with help of the PAST software [11]. For the distinction of isolates, the presence or absence of peaks was considered with or without taking peak intensities into account. The results were compared with the results of amplified fragment length polymorphism (AFLP) typing.

Experiments were performed as follows: Isolates were grown for 24 h, and four single colonies were selected, spotted and overlaid with CHCA-based matrix. The replicate colony samples were measured on a VITEK MS instrument set to the research use only configuration. The mass range was 2000 to 20,000 Da, using standard settings for routine identification. The spectra were processed and imported into the SARAMIS database. Consensus spectra were generated for each isolate, retaining only peaks that were present in at least three out of the four spectra. Sixty-one peaks were common to all consensus spectra of the 52 *E. coli* isolates tested. These peaks were excluded from the analyses, as were peaks that were easily determined to be due to multiply charged peptides.

Analyses were carried out with the help of PAST3, taking either the presence or absence of peaks alone into account, or in combination with relative peak intensities which had first been logarithmically transformed. The resolution was improved when taking the logarithmically transformed peak intensities into account, compared with using the presence or absence of peaks for analysis alone. However, whilst this was the case for the transformed peak intensities, using the relative peak intensities without transformation resulted in poor resolution with hardly any clustering.

Four clusters were obtained which were largely in agreement with amplified fragment length polymorphism typing when using MALDI-TOF MS typing, although with lower resolution. Furthermore, MALDI-TOF MS typing could correctly identify the outbreak isolates included in the study [5].

Christner et al. evaluated a marker peak-based strategy for MALDI-TOF MS typing of a Shiga toxigenic *Escherichia coli* outbreak strain. Data for strain distinction were acquired using a Bruker MALDI biotyper and analysed with the help of MALDIquant [12]. Two peaks were chosen as outbreak strain biomarkers.

Experiments were performed as follows: Study isolates were grown overnight and spotted directly in triplicate, as well as subjected to ethanol/formic acid extraction and spotted in triplicate. This was carried out three times from independent cultures. Spots were overlaid with saturated CHCA solution in 50% acetonitrile and 2.5% trifluoroacetic acid. Spectra were recorded in a Bruker MALDI biotyper in the range of 2000 to 20,000 Da. The spectra were internally calibrated with the help of known m/z values of conserved ribosomal proteins and then processed further using MALDIquant. Pre-outbreak spectra that had been recorded as single spectra for routine bacterial identification using the direct streak method were used for comparison after processing them in the same way as the newly acquired spectra.

Peaks that occurred in at least eight out of the nine spectra generated by either the direct deposit or extraction method were merged into a combined peak list and compared against 150 *E. coli* pre-outbreak spectra. The discriminatory mass peaks were determined by using a consensus peak list of a selected outbreak isolate for each of the two methods. Applying an m/z tolerance of 400 ppm, six discriminatory peaks could be detected, out of which two peaks (6711 m/z and m/z 10,883) were chosen as outbreak strain biomarkers.

Evaluation was performed with 293 clinical isolates, out of which 104 were outbreak isolates as determined by PCR genotyping. With the extraction method, 99.7% of all isolates were classified correctly, and with the direct deposit method, 99% of all isolates were classified correctly [13].

### 2.5. MALDI-TOF MS Typing of Serratia marcescens and Citrobacter freundii

Rödel et al. investigated the discriminatory power of MALDI-TOF MS for measuring hospital-outbreak related isolates of *Serratia marcescens* (33 isolates) and *Citrobacter freundii* (23 isolates). Data for strain distinction were acquired on a bioMérieux VITEK MS instrument and analysed using the relative taxonomy analysis tool of the SARAMIS premium software. The results were compared with the results of allele typing of the core genome of the isolates of each species.

Experiments were performed as follows: Single colonies of each bacterial isolate were spotted onto the target in triplicate and overlaid with CHCA-based matrix (bioMérieux). Spectra were acquired in the research modus with the mass range being between 3000 and 20,000 Da and imported to the SARAMIS^TM^ database. The three single spectra of each isolate were used to build a consensus spectrum, considering only the mass signals that were present for all three replicates. A mass accuracy of 800 ppm was applied for peaks considered to be identical. Fine tuning of the laser of the VITEK MS instrument was performed fortnightly by the technical service of bioMérieux, and same age bacterial cultures were always used for measurement.

For final analysis, a mass range from 3000 to 15,000 Da was taken into account, not considering the mass signal intensities. Dendrograms were generated using the relative taxonomy analysis tool of the SARAMIS premium software.

Although the discriminatory power of WGS could not be reached using MALDI-TOF MS typing, congruence was found, and the authors suggest that it may be used as a first-line sub-typing tool [3].

### 2.6. MALDI-TOF MS Typing of Enterococcus faecium

Holzknecht et al. typed 55 WGS sequenced vancomycin-resistant *Enterococcus faecium* isolates collected in an outbreak situation using MALDI-TOF MS. Data for strain distinction were acquired on a Bruker MALDI-TOF MS instrument and analysed with the FlexAnalysis software (Bruker). Nine peaks in the mass range from 3341 to 7848 Da were chosen for discrimination. The results were compared with the results of single nucleotide polymorphism analysis of the respective whole genome sequences.

The experiments were performed as follows: Isolates were grown overnight for the MALDI-TOF MS experiments. Three ethanol/formic acid extractions were carried out, each of which was spotted thrice. The spots were overlaid with CHCA-based matrix (Bruker Daltonik GmbH, Bremen, Germany) and measured three times in a Bruker MALDI-TOF MS instrument, resulting in 27 spectra per isolate. In addition to the standard calibration, an internal calibration at 6341.4 Da was performed. Only peaks that were consistent in all three extractions were included for further analysis.

Nine peaks of singly charged peptides that were reproducibly present in the range from 3341 to 7848 Da in some but not all of the isolates investigated were chosen for discrimination.

Whilst the method could differentiate three outbreak strains from each other, non-outbreak isolates could not reliably be differentiated from outbreak isolates, limiting the method’s usefulness for outbreak management [14].

### 2.7. MALDI-TOF MS Typing of Streptococcus agalactiae

Rothen et al. tested the ability of MALDI-TOF MS to type *S. agalactiae.* Data for strain distinction were obtained using whole genome sequences and in-house software to calculate the masses of ribosomal subunit proteins predicting MALDI peaks. This in silico-generated database was validated with isolate spectra acquired using a Shimadzu AXIMA MALDI instrument, or, for comparison, a Bruker Microflex instrument. The results were compared with the results of core genome phylogenetic analysis of the respective whole genome sequences.

Experiments were performed as follows: The masses of ribosomal subunit proteins from 796 highly diverse mainly publicly available whole genome sequences of *Streptococcus agalactiae* were calculated, making use of a python bioinformatics pipeline. Sixty-two unique profiles could be identified, using 28 reproducibly measurable ribosomal subunit proteins in the mass range from 4425 to 19,293 Daltons. Core genome phylogenetic analysis of the whole genome sequences was performed, and *S. agalactiae* isolates with the same ribosomal subunit protein profiles correlated well with the clustering.

A total of 256 isolates were grown overnight, sub-cultured and regrown before protein extraction. Samples were washed repeatedly in a buffer, bacterial cells were disrupted using a bead beater and fragments smaller than 3000 Daltons were then removed by filtration. The samples were reconstituted, spotted in quadruplicate and overlaid with a sinapinic acid-based matrix (10 mg sinapinic acid in 60% acetonitrile, 40% ddH2 O and 1% TFA).

Measurements were carried out in a Shimadzu AXIMA MALDI instrument, except for eight isolates which were measured externally using a Bruker Microflex instrument. The mass range was 4000 to 25,000 Daltons and the pulsed extraction was optimised at 20,000 Daltons. The ion gate was set at 3950 Daltons. The spectra were processed and an internal calibration was carried out with a mass tolerance of 800 ppm, using known masses.

Mass spectrum peaks were queried against the in silico-predicted mass alleles and assigned to ribosomal subunit protein profiles.

As a proof of principle, 37 *S*. *agalactiae* isolates with whole genome sequences available were measured using MALDI-TOF MS. These included 11 isolates from humans, 16 isolates from camels, two from cattle and eight isolates from fish which were measured in an external laboratory with different MALDI-TOF equipment. All but one isolate of human origin were assigned correctly to their respective ribosomal subunit protein profile.

An additional 219 isolates that did not have whole genome sequences available were subsequently measured. These included 154 isolates from humans, 63 isolates from camels and two isolates from cattle. All but three isolates from humans could be assigned to a ribosomal subunit protein profile [15].

### 2.8. MALDI-TOF MS Typing of Tenacibaculum maritimum

Bridel et al. looked at the potential of MALDI-TOF MS to type the marine fish pathogen *Tenacibaculum maritimum.* The global genomic diversity of the bacterial species was investigated by comparing the genomes of 25 isolates from various geographical and host fish origins. MALDI data were acquired using a Bruker MALDI biotyper and processed using R packages dedicated to mass spectrometry. Detected mass peaks from isolates with available genome sequences were assigned to ribosomal proteins. Nine such peaks that were subject to variability were chosen as polymorphic biomarkers to subdivide the isolates into MALDI types and MALDI groups. The resulting clusters were compared with whole genome alignment. The detectability of the biomarkers was validated using 111 field isolates.

The experiments were performed as follows: Bacterial samples were prepared using an extraction procedure as well as a direct streak method for comparison. A CHCA-based matrix (CHCA in 50% acetonitrile and 2.5% trifluoroacetic acid) was used. The spectra were acquired in a Bruker MALDI biotyper, with a mass range of 2000 to 20,000 Daltons, applying a mass tolerance of 300 parts-per-million (ppm) for external calibration. For each isolate, spectra were recorded in quadruplicate thrice.

The genome sequence data were used to assign several mass peaks detected in the MALDI-TOF MS spectra to ribosomal proteins. Ribosomal proteins with conserved masses were used for internal calibration and species assignment, and ribosomal proteins with variable masses served as biomarkers for typing. Nine polymorphic biomarkers ranging from 6600 to 13,200 Da were chosen and used to match with peaks detected in MALDI-TOF MS spectra. A peak was considered identical if the difference amounted to less than 700 ppm.

The approach exhibited a high success rate for the extraction method, where the chosen polymorphic biomarkers could be detected in excess of 99.5% in spectra acquired from 111 field isolates [4].

## 3. Conclusions

MALDI-TOF MS is routinely used in clinical laboratories for identification of bacterial species. It would be of great benefit to also be able to also use MALDI-TOF MS for the identification of bacterial strains that are of clinical relevance [3].

The studies reviewed in this paper used a variety of acquisition and processing methods, as well as genotyping reference methods for strain distinction, and reported different levels of success. As we are presented with a great number of variables, it is challenging to determine what makes MALDI-TOF MS typing of bacterial strains work. Table 1 reviews a few methods and the levels of success shared by multiple studies. 

Multiple spectra of each isolate recorded either during the same run or different runs were often combined before analysing mass spectral differences for strain distinction [1,3,5,7,8,9,10,13,14].

In general, any mass peaks chosen for strain discrimination were below 10,000 m/z [1,2,9,14] or no higher than 15,000 m/z [3,4,10,13]. There was one exception where mass peaks up to 19,293 Daltons were used for strain discrimination, and measures were taken to improve the quality of higher mass peaks. These included protein extraction, the removal of mass molecules below 3000 Daltons, the enabling of the low mass ion gate at 3950 Daltons, the optimisation of pulsed extraction at 20,000 Daltons and the use of a sinapinic acid-based matrix [15].

Ideally, the same MALDI-TOF MS data used for bacterial identification can also be used for isolate typing, allowing for on-site monitoring of outbreak strains [1]. However, to increase resolution, an extraction step has often been performed for MALDI-TOF MS typing of bacterial isolates [4,7,9,13,14,15]. In order to increase mass accuracy, internal calibration may be performed in addition to external calibration, along with the application of a low mass tolerance [2,4,13,14,15].

As successful MALDI-TOF strain typing may depend on the lineages investigated [6], it would be beneficial to be able to assess feasibility before embarking on big lab projects. Ribosomal proteins account for about 50% of the peaks detected in MALDI-TOF MS spectra. The use of WGS data for in silico calculation of ribosomal masses may help determine the feasibility of MALDI-TOF MS typing of bacterial isolates at an early stage. Polymorphic ribosomal proteins may be used for strain distinction, and the conserved ones may be used for internal calibration to increase mass accuracy [4,15]. The use of previously recorded spectra of isolates circulating in a certain setting may also help the early determination of the MALDI-TOF MS typeability of a particular bacterial strain [13].

## Figures and Tables

**Table 1:** Methods shared amongst multiple studies and levels of success of each study.

Reference	Bacterial Species Investigated	Combining of MALDI Spectra for Data Analysis	Protein Extraction of Bacterial Samples	Internal Calibration in Addition to External Calibration	Level of Success
[1]	*Klebsiella pneumoniae*	Yes	No	No	High
[2]	*Staphylococcus aureus*	No	No	Yes	Intermediate
[3]	*Serratia marcescens*/*Citrobacter freundii*	Yes	No	No	Intermediate
[4]	*Tenacibaculum maritinum*	No	Yes	Yes	High
[5]	*Escherichia coli*	Yes	No	No	High
[6]	*Staphylococcus aureus*	No	No	No	High
[7]	*Staphylococcus aureus*	Yes	Yes	No	High (93% in comparison with reference methods)
[8]	*Klebsiella* sp.	Yes	No	No	Low
[9]	*Klebsiella pneumoniae*	Yes	Yes	No	Intermediate/low (low resolution of reference method)
[10]	*Clostridium difficile*	Yes	No	No	High/intermediate
[13]	*Escherichia coli*	Yes	Yes	Yes	High (99.7% for extraction method and 99% with direct deposit method)
[14]	*Enterococcus faecium*	Yes	Yes	Yes	Intermediate/low
[15]	*Streptococcus agalactiae*	No/in silico calculation of reference spectra	Yes	Yes	High
